# Two Weeks of Metformin Treatment Enhances Mitochondrial Respiration in Skeletal Muscle of AMPK Kinase Dead but Not Wild Type Mice

**DOI:** 10.1371/journal.pone.0053533

**Published:** 2013-01-14

**Authors:** Jonas M. Kristensen, Steen Larsen, Jørn W. Helge, Flemming Dela, Jørgen F. P. Wojtaszewski

**Affiliations:** 1 Section of Molecular Physiology Group, August Krogh Centre, Department of Nutrition, Exercise and Sport Sciences, University of Copenhagen, Denmark; 2 Xlab, Center of Healthy Aging, Department of Biomedical Sciences, Faculty of Health Sciences, University of Copenhagen, Denmark; 3 Section of Molecular Diabetes & Metabolism, Endocrinology Research Unit, Institute of Clinical Research University of Southern Denmark, Denmark; Tohoku University, Japan

## Abstract

Metformin is used as an anti-diabetic drug. Metformin ameliorates insulin resistance by improving insulin sensitivity in liver and skeletal muscle. Reduced mitochondrial content has been reported in type 2 diabetic muscles and it may contribute to decreased insulin sensitivity characteristic for diabetic muscles. The molecular mechanism behind the effect of metformin is not fully clarified but inhibition of complex I in the mitochondria and also activation of the 5′AMP activated protein kinase (AMPK) has been reported in muscle. Furthermore, both AMPK activation and metformin treatment have been associated with stimulation of mitochondrial function and biogenesis. However, a causal relationship in skeletal muscle has not been investigated. We hypothesized that potential effects of *in vivo* metformin treatment on mitochondrial function and protein expressions in skeletal muscle are dependent upon AMPK signaling. We investigated this by two weeks of oral metformin treatment of muscle specific kinase dead α_2_ (KD) AMPK mice and wild type (WT) littermates. We measured mitochondrial respiration and protein activity and expressions of key enzymes involved in mitochondrial carbohydrate and fat metabolism and oxidative phosphorylation. Mitochondrial respiration, HAD and CS activity, PDH and complex I-V and cytochrome c protein expression were all reduced in AMPK KD compared to WT tibialis anterior muscles. Surprisingly, metformin treatment only enhanced respiration in AMPK KD mice and thereby rescued the respiration defect compared to the WT mice. Metformin did not influence protein activities or expressions in either WT or AMPK KD mice.

We conclude that two weeks of *in vivo* metformin treatment enhances mitochondrial respiration in the mitochondrial deficient AMPK KD but not WT mice. The improvement seems to be unrelated to AMPK, and does not involve changes in key mitochondrial proteins.

## Introduction

Metformin is worldwide the most prevalently used drug to treat type 2 diabetes and have been utilized in the clinic for more than five decades to improve insulin sensitivity [1], [2]. Type 2 diabetes mellitus is associated with decreased insulin sensitivity in liver and skeletal muscle. It has been reported that diabetic muscles have reduced mitochondrial content as well as protein expressions and activities and this may contribute to skeletal muscle insulin resistance [3]–[5]. However, several other studies have not confirmed these findings [6]–[10]. The molecular mechanisms behind the effects of metformin are not fully clarified. Although inhibition of complex I in the mitochondrial electron transport chain (ETC) in cells, isolated mitochondria and muscle homogenate has been reported [11]–[15] consensus is not reached [16], [17]. In addition, metformin-induced activation of the 5′-AMP activated protein kinase (AMPK) has been shown in rodent muscles [18]–[20], and in human skeletal muscles from patients with type 2 diabetes [21], [22]. AMPK is a heterotrimeric kinase with a catalytic α subunit and regulatory β and γ subunits. AMPK activity is enhanced when the AMP∶ATP and/or ADP∶ATP ratio within the cell increases. Upon activation AMPK inhibits ATP consuming pathways and enhances ATP producing pathways and thereby acts as a cellular fuel gauge [23]–[25]. Stimulation of AMPK by metformin is not via direct interaction [18], [26], [27]. Instead activation has been suggested to be linked to the inhibition of mitochondrial complex I, that may lead to a disturbance in cell energy balance and thus AMPK activation [13], [27], [28], although other studies suggest that the metformin-induced activation is not dependent of such changes in the energy status of the cell [26], [29].

Numerous studies using transgenic models and pharmacological AMPK activation have documented a role for AMPK in regulating mitochondrial function and biogenesis [30]–[34]. On the other hand it is sparse with reports on the potential effects of metformin treatment on mitochondrial function besides the above mentioned studies in regard to complex I inhibition. Some studies suggest that metformin also affects mitochondrial oxidative stress by decreasing ROS production [17], [35], [36] and interestingly, an *in vitro* study with endothelial cells has shown AMPK dependent metformin-induced promotion of mitochondrial biogenesis [37]. In muscles, stimulation of PGC-1α, a key regulator of mitochondrial biogenesis [38], has been reported both *in vitro* and *in vivo* after long term metformin treatment [20], [39], [40] together with increased activities and protein expressions of mitochondrial marker enzymes [20], [40].

Even though metformin treatment and AMPK signaling both have been associated to mitochondrial regulation no study has to our knowledge investigated a possible causal relationship in skeletal muscle. Thus, we hypothesized that potential effects in skeletal muscle of metformin treatment *in vivo* on mitochondrial function and protein expressions are dependent upon AMPK signaling. Therefore, we genetically investigated the role of AMPK for metformin to stimulate mitochondrial function and biogenesis in skeletal muscle by use of the muscle specific kinase dead α_2_(KD)AMPK mice.

## Methods

### Animals

Experiments were approved by the Danish Animal Experimental inspectorate and complied with the European Convention for protection of vertebra animals used for scientific purposes. The mice used in these experiments were muscle specific kinase dead α_2_ (KD) AMPK mice that have been described previously [41]. The female mice were 22–30 weeks old littermates on a C57BL/6 background. Wild type (WT) littermates were used as controls for the genetic model.

### Treatment

All animals were kept on a 12 h/12 h light/dark cycle with unlimited access to standard rodent chow food and water. Cages were provided with bedding, shelter and bit stick.

WT and KD mice were divided into two groups; one treated with metformin and the other given saline. Metformin (2*150 mg*kg^−1^*day^−1^) and saline solutions were administered by gavage twice daily in the morning and afternoon for two weeks. The last dosage of metformin/saline was given the afternoon before the experimental day. On the experimental day mice were anesthetized by intraperitoneal injection of pentobarbital (0.1 mg*g body wt^−1^). TA muscles were isolated from both legs. One portion of TA was put in a relaxing buffer (content is described below) and used for high resolution respirometry, while the rest and the whole other TA was frozen in liquid nitrogen and stored at −80°C for later analysis.

### Preparation of permeabilized skeletal muscle fibers for high resolution respirometry

The TA muscle tissue was subjected to mechanical dissection with sharp forceps in relaxing buffer on ice. The relaxing buffer contained 2.77 mM CaK2EGTA, 7.23 mM K2EGTA, 20 mM imidazole, 20 mM taurine, 6.56 mM MgCl2, 5.77 mM ATP, 15 mM phosphocreatine, 0.5 mM dithiothreitol, and 50 mM K-MES, pH 7.1. Fiber bundles were permeabilized by gentle agitation for 30 min at 4°C in the relaxing solution, supplemented with 50 µg/ml saponin. Fiber bundles were then washed in ice-cold respiration medium (content described below) 2×10 minutes at 4°C during gentle agitation.

### High-resolution respirometry

Respiration was measured at 37°C in a high resolution respirometer (Oroboros, Oxygraph; Innsbruck, Austria). The respiration medium consisted of 110 mM sucrose, 60 mM K-lactobionate, 0.5 mM EGTA, 1 g/l BSA essentially fatty acid free, 3 mM MgCl2, 20 mM taurine, 10 mM KH2PO4, 20 mM K-HEPES, pH 7.1. The software DatLab (Oroboros) was used for data analysis. The use of permeabilized fibers provides the possibility to study mitochondria “in situ” in the cell, and very small muscle samples (2–4 mg) are needed to study mitochondrial respiration.

Mitochondrial respiration protocol: In order to avoid any potential oxygen diffusion limitation all experiments were conducted after hyper-oxygenation (450 nmol O2/ml). Substrate and inhibitor were added consecutively, one protocol was employed, made in duplicate. State 2 respiration was assessed with malate (2 mM) a complex I substrate. State 3 respiration was reached with ADP (5 mM) and subsequently glutamate (10 mM) another complex I substrate. This was followed by succinate (10 mM) a complex II substrate, resulting in parallel electron input to complex I and II. Cytochrome c (10 µM) was added to control for outer mitochondrial membrane integrity. Finally rotenone (0.5 µM) was added to inhibit complex I. Malate and glutamate are providing NADH for complex I and succinate is providing FADH for complex II.

### Muscle lysate preparation used for Western blot analyzes and homogenates used for Citrate Synthase (CS) and 3-Hydroxyacyl-CoA Dehydrogenase activity (HAD) activity

For extraction of proteins the whole muscle were homogenized in ice cold buffer (10% glycerol, 20 mM sodium pyrophosphate, 150 mM sodium chloride, 50 mM HEPES, 1% NP-40, 20 mM β-glycerophosphate, 10 mM sodium fluoride, 2 mM PMSF, 1 mM EDTA, 1 mM EGTA, 10 µg/ml aprotinin, 10 µg/ml leupeptin, 2 mM Sodium orthovanadate, 3 mM benzamidine, pH 7.5) by a tissue lyser for 2*1 min at 30 Hz (Tissue Lyser II, Qiagen Retch, Germany). Muscle homogenates were rotated end over end at 4°C for 1 hour, after which they were centrifuged for 20 min at 15.000 g. The supernatants were harvested as the muscle lysate and stored at −80°C. Total lysate protein content was determined by the bicinchoninic acid method (Pierce Biotechnology, Inc., IL, USA). Muscles used for HAD and CS activity were homogenized in ice cold buffer (0.3 M potassium phosphate pH = 7.7, 0.05% BSA) by a tissue lyser for 2*1 min at 30 Hz (Tissue Lyser II, Qiagen Retch, Germany). HAD and CS activity were measured on the homogenate as described below.

### SDS page and Western blot analyzes

Lysate proteins were separated by SDS-PAGE on self cast Tris-HCl (7–12%) gels and by semidry blotting transferred to a PVDF membrane (Immobilon Transfer Membranes; Millipore, Bagsværd, Denmark). The membrane was blocked in a washing buffer (10 mM Tris-base, 150 mM NaCl and 0.25% Tween 20) containing low fat milk protein milk or BSA solution and afterwards probed with primary antibodies and appropriate secondary antibodies (see below). Protein bands were visualized using a Kodak Image Station (2000 MM, Kodak, N.Y., USA) after probing with enhanced chemiluminescence (ECL, Millipore, Mass, USA). Bands were quantified using Kodak 1D 3.6 software (Kodak, N.Y., USA).

#### Antibodies used for detection of specific phosphorylation sites and protein expressions

Mitochondria complex I protein expression: Complex I, Molecular Probes, Invitrogen (#A31857); CA, USA. Mitochondria complex II protein expression: Complex II, Molecular Probes, Invitrogen (#A11142); CA, USA. Mitochondria complex III protein expression: Complex III, Molecular Probes, Invitrogen (#A21362); CA, USA. Mitochondria complex IV protein expression: Anti-Oxphos Complex IV Subunit I Monoclonal Antibody, Invitrogen (#459600), CA, USA. Mitochondria Complex V: F1-ATPase, Santa Cruz Biotechnology (#sc-16689); CA, USA. Cyt C protein expression: Anti-Cytochrome C monoclonal antibody: BD Biosciences(#556433); NJ, USA. Pyruvate Dehydrogenase (PDH) subunit E1α (PDH-E1α) protein expression and PDH-E1α site 1 (Ser293) and Site2 (Ser300) phosphorylations were determined as described previously [42]. Acetyl-CoA carboxylase (ACC) phosphorylation at Ser^221^: anti-phospho-Acetyl CoA Carboxylase (Ser^79^), Upstate Biotechnology, MA, USA (#07-303); anti AMPK α1 and α2 protein expressions: anti AMPK α1 and α2 specific antibodies raised in sheep as previously described [43] was kindly donated by Hardie DG, University of Dundee, Scotland, UK.

Secondary HRP-conjugated antibodies used were from Dako (Glostrup, Denmark).

### Citrate Synthase (CS) and 3-Hydroxyacyl-CoA Dehydrogenase activity (HAD) activity

CS and HAD activity was measured in a reaction coupled to conversion of NAD to NADH by spectrophotometric determination of NADH changes at 340 nm at 37°C, pH 7.0, in muscle homogenates, using an automatic analyzer (Hitachi automatic analyzer 912; Boehringer Mannheim, Ingelheim, Germany). CS activity was measured with acetyl-CoA and oxaloacetate as substrate and HAD measured with acetoacetyl-CoA as substrate [44]

### Statistics

Differences between groups were evaluated by Two-Way ANOVA or Students t-test. Significant main effects or interactions were further analyzed by Student-Newman-Keuls method as post hoc test. Differences between groups were considered statistically significant when P<0.05. All data are expressed as means ± S.E.M.

## Results

### Decreased state 3 mitochondrial respiration in AMPK KD compared to WT muscle is normalized after metformin treatment

**Figure 1:**
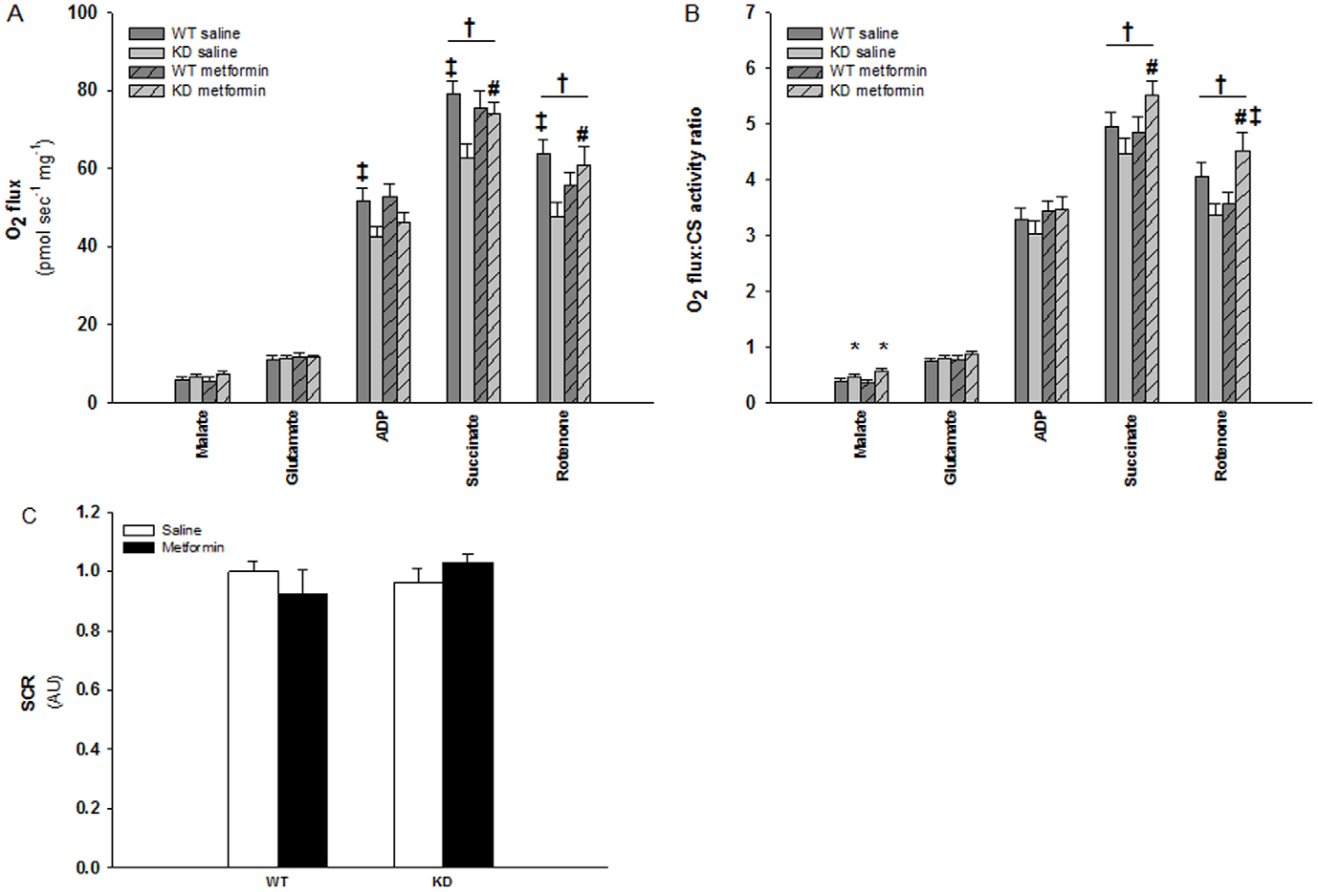
Figure 1A+B: Mitochondrial respiratory capacity in TA muscles from WT and KD mice treated with metformin or saline for two weeks. A: Mitochondrial respiration (O_2_ flux) related to milligram muscle. B: Mitochondrial respiration (O_2_ flux) related to milligram muscle and expressed relative to mitochondrial content (CS activity). Dark grey bars shows results in TA muscles from saline treated WT mice. Light grey bars shows results in TA muscles from saline treated KD mice. Dark grey bars with slashes shows results in TA muscles from metformin treated WT mice. Light grey bars with slashes shows results in TA muscles from metformin treated KD mice. ‡: Indicate significant difference between genotype within intervention (P<0.05). #: Indicate significant difference between interventions in AMPK KD mice (P<0.05). †: Indicate significant interaction between genotype and intervention (P<0.05). *: Indicate significant main effect of genotype (P<0.05). n = 11–14. Values are means ± S.E.M. Figure 1C: Substrate control ratio (SCR) is calculated as state 3 respiration with complex I and II linked substrates divided by state 3 respiration with complex I linked substrates. White bars shows results in TA muscles from saline treated mice. Black bars shows results in TA muscles from metformin treated mice. n = 11–14. Values are means ± S.E.M.

Mitochondrial respiration was significantly decreased in TA muscles of saline treated AMPK KD compared to WT mice when measured per milligram of muscle (Fig. 1A). There was no difference in state 2 respiration (Fig. 1A, Malate) but state 3 respiration was reduced by ∼20% (P<0.05) with electron flux through complex I (Fig. 1A, Malate, Glutmate and ADP) and by ∼20% (P<0.01) with electron flux through complex I+II (Fig. 1A, Malate, Glutmate, ADP and Succinate) in saline treated AMPK KD compared to WT mice. Rotenone was added to inhibit complex I, but this inhibition did not affect the above described differences, i.e. ∼20% reduced respiration in AMPK KD compared to WT muscle (Fig. 1A, Rotenone). Furthermore, we calculated the substrate control ratio (SCR) for succinate (state 3 respiration with complex I and II linked substrates and state 3 respiration with complex I linked substrates). There was no significant effect of genotype or treatment in SCR, but a tendency was seen (P = 0.062) for the treatment in the KD mice, indicating that the difference in respiration could be explained by a different complex II linked respiration (Fig. 1C)

Two weeks of oral metformin treatment did not influence mitochondrial respiration in WT muscles but stimulated state 3 respiration in KD muscles through complex II (Fig. 1A, P<0.05), supported by the tendency found in SCR in the KD mice after metformin treatment. This implies that the defect in electron flux through complex II in KD muscles were rescued compared to WT muscles (Fig. 1A, P<0.05). These findings indicate a defect in KD mice mitochondrial complex II or downstream of complex II in the ETC that is rescued upon metformin treatment. Mitochondrial membrane integrity was controlled by addition of cytochrome c (results not shown).

When mitochondria O_2_ flux rates were expressed relative to mitochondrial content, i.e. CS activity, there were no longer significant differences in state 3 respiration with electron flux through complex I (Fig. 1B, Malate, Glutamate and ADP) and II (Fig. 1B, Malate, Glutamate, ADP and Succinate). This indicates that the observed difference in mitochondria respiration between WT and KD muscles in saline treated mice (Fig. 1A) is due to decreased mitochondrial content in KD muscles. There was a main effect of genotype in state 2 respiration after normalization to CS activity (Fig. 1B, Malate).

However, the different effect of metformin treatment in WT and KD muscles persisted after adjusting respiration to mitochondrial content (Fig. 1B, Succinate). I.e. that metformin-induced stimulation of mitochondrial respiration with ∼30% in KD mice probably was caused by enhancement of mitochondrial function and not mitochondrial content.

### Reduced protein expressions of mitochondria respiration chain complex I-V and cytochrome C in AMPK KD compared to WT muscles but no effect of metformin treatment

**Figure 2:**
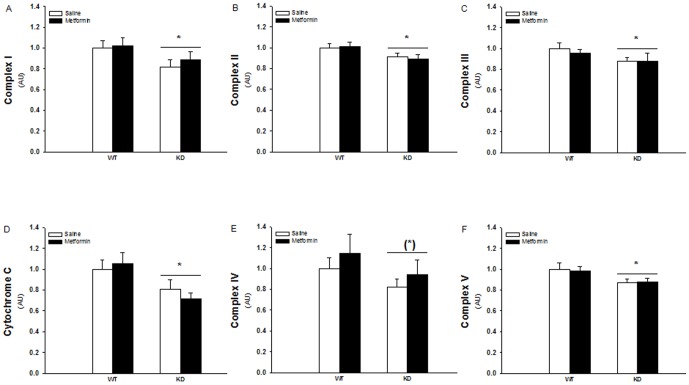
Protein expressions of Complex I–V and Cytochrome C of the mitochondria electron transport chain in TA muscles from WT and KD mice treated with metformin or saline for two weeks. White and black bars shows results from saline treated and metformin treated mice respectively. A: Complex I protein expression. B: Comp lex II protein expression. C: Complex III protein expression. D: Cytochrome C protein expression. E: Complex IV protein expression. F: Complex V protein expression. *: Indicates significant difference between genotype (P<0.05). (*): P = 0.059. N = 12–17. Values are S.E.M.

**Figure 3:**
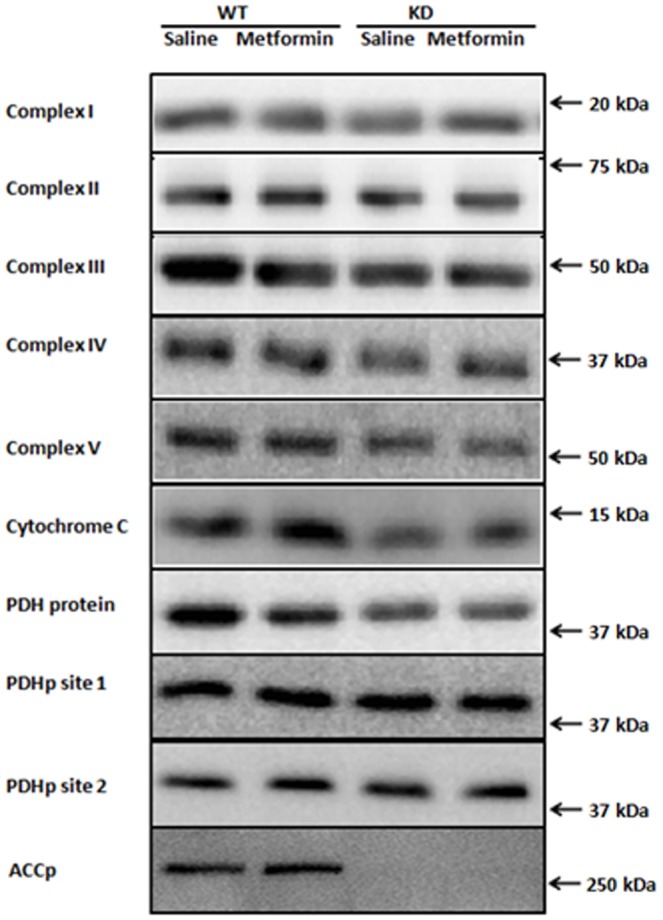
Representative Western blots of Complex I–V, Cytochrome C, Pyruvate Dehydrogenase (PDH) subunit E1α protein, PDH site1 and PDH site2 phosphorylation and ACC^Ser211^ phosphorylation.

To elucidate whether the observations on mitochondrial respiration in regard to the role of AMPK and metformin treatment are reflected in the different protein complexes of the mitochondrial ETC, we choose to measure protein expression on a subset of proteins representing complex I–V and cytochrome c in the ETC (Fig. 2A–2F and Fig. 3). AMPK KD mice had generally decreased protein expression levels of these complexes and cytochrome c in the range of 10–25% (Fig. 2A–2F, P<0.05). Metformin treatment did not change protein expressions in either WT or KD muscles.

### Reduced PDH expression in AMPK KD compared to WT muscles but no effect of metformin treatment on PDH expression or phosphorylation

**Figure 4:**
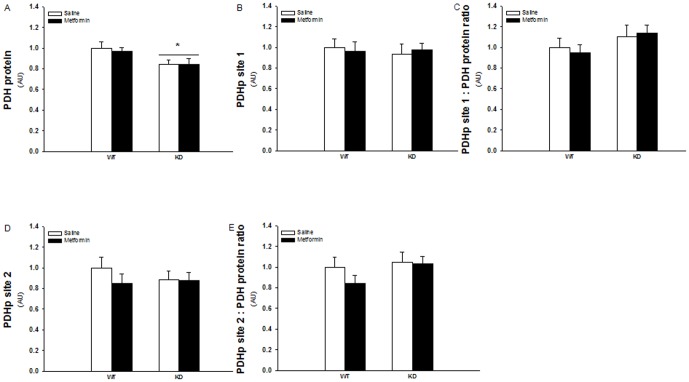
PDH site 1 and 2 phosphorylation and protein expression in TA muscles from WT and KD mice treated with metformin or saline for two weeks. White and black bars shows results from saline treated and metformin treated mice respectively. A: PDH protein expession. B: PDH site 1 phosphorylation. C: PDH site 1 phosphorylation related to PDH protein expression. D: PDH site 2 phosphorylation. E: PDH site 1 phosphorylation related to PDH protein expression. *: Indicates significant difference between genotype (P<0.05). N = 12–17. Values are S.E.M.

The pyruvate dehydrogenase complex (PDH) enzyme catalyzes conversion of pyruvate to acetyl-CoA and is a key regulator of carbohydrate-derived substrate into the tricarboxylic acid cycle (TCA cycle). The PDH protein expression was decreased ∼15% in muscles from AMPK KD mice compared to WT muscles, but the expression was not regulated by metformin treatment in either of the genotypes (Fig. 4A) Phosphorylation at site 1 and site 2 on the PDH enzyme is believed to reduce its activity [45] but none of these sites were significantly affected by genotype or metformin treatment (Fig. 4B–E and Fig. 3).

### Reduced HAD and CS activity in AMPK KD compared to WT muscles but no effect of metformin treatment

**Figure 5:**
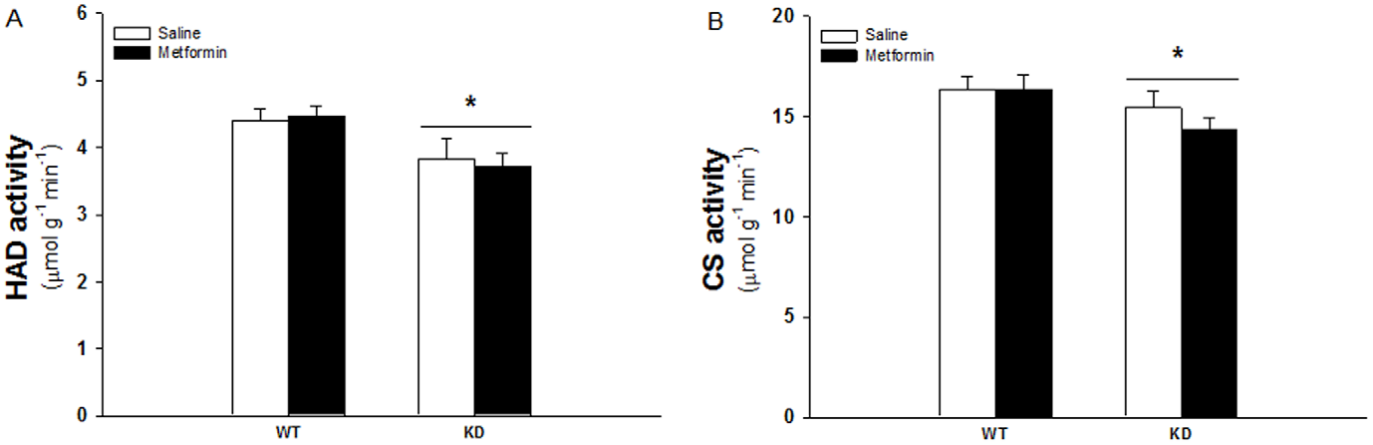
HAD and CS activity in TA muscles from WT and KD mice treated with metformin or saline for two weeks. White and black bars shows results from saline treated and metformin treated mice respectively. A: HAD activity. B: CS activity. *: Indicates significant difference between genotype (P<0.05), N = 12–17. Values are S.E.M.

HAD and CS activity was measured as key enzymes in the β-oxidation and TCA cycle respectively. HAD activity was repressed about 15% in AMPK KD muscles but there was no effect of metformin treatment in either of the genotypes (Fig. 5A). Similarly CS activity was repressed about 10% in AMPK KD muscles and not influenced by metformin treatment (Fig. 5B).

### Evaluation of remaining endogenous AMPK α subunit expression and activity in muscles from AMPK-KD mice

**Figure 6:**
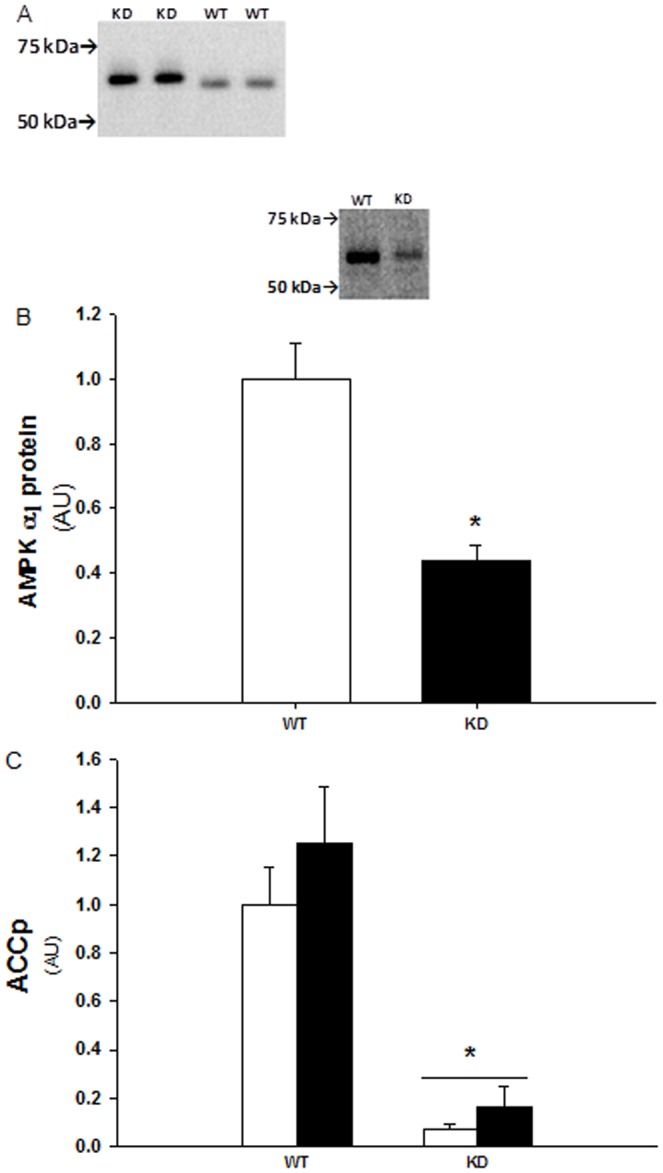
Protein expression of AMPKα subunits and ACC Ser^221^ phosphorylation. A: Representative Western blot of AMPK α_2_ in AMPK WT and KD muscles. The absence of a detectable band at the expected molecular weight of ∼63 kDa in KD mice muscles indicates a strongly reduced endogenous α_2_ protein expression. Instead the slightly heavier band in KD muscles is detection of the myc-tag transgenic α_2_ protein. B: α_1_ protein expression in WT and AMPK KD muscles. *: Indicates significant difference between genotype, P<0.001, n = 10. Values are means ± S.E.M. C: ACC Ser^221^ phosporyaltion in AMPK WT and KD muscle lysates (TA) from chronic saline (white bars) and metformin (black bars) treated mice. *: Indicates significant difference between genotype, P<0.001, n = 11–14. Values are means ± S.E.M.

The AMPK-KD mice used in the present study are derived from over-expressing a kinase dead Lysine^45^ – Arginine mutant of the 5′AMP activated protein kinase (AMPK) α_2_ protein, driven by the heart and skeletal muscle specific creatine kinase promoter [41]. As shown in figure 6A, a Western blot of AMPK α2 in muscles from WT and AMPK KD mice, there is no detectable band at the expected molecular weight of the endogenous α_2_ protein in the AMPK KD muscles. Due to a myc-tag, the transgenic α_2_ protein can be detected as a slightly heavier and stronger band compared to the WT α_2_ protein which is detected at ∼63 kDa. Furthermore over-expression of the kinase dead α_2_ protein results in a ∼50% reduction of α1 protein content as shown in figure 6B. As an indirect measure of both α1 and α2 associated activity we evaluated Ser212 phosphorylation of ACC, an AMPK substrate (Fig. 3+6C). Based on the ACC phosphorylation total AMPK activity in muscles from AMPK KD mice are reduced to ∼10% compared with muscles from wild type mice. Furthermore, there was no significant effect of the chronic metformin treatment on ACC phosphorylation in wild type or KD muscles. Notice that the last dose of metformin/saline was given the afternoon before muscles were isolated from the anesthetized mice. Consequently, the graphic illustration represents the chronic and not the acute effect of metformin on ACC phosphorylation.

## Discussion

The aim of the present study was to investigate potential effects of two weeks of *in vivo* metformin treatment on mitochondrial function as well as activity and expression of mitochondrial proteins. We hypothesized that these assumed metformin effects in skeletal muscle would be dependent upon AMPK signaling.

To our knowledge we are the first to elucidate the consequence of genetically induced AMPK down regulation on mitochondrial respiration after metformin treatment in skeletal muscle. We observed decreased mitochondrial respiration related to electron flux through mitochondrial complex I and II in TA muscles from AMPK KD mice. Metformin treatment did not affect mitochondrial respiration or mitochondrial protein content and activity in WT muscles. However, metformin stimulated mitochondrial respiration in KD muscles. Thus, mitochondrial respiration via electron flux through complex II and/or downstream of complex II in the ETC was enhanced in KD muscles. Electron flux through complex I was unaffected by metformin. Since neither of the mitochondrial marker enzymes representing the ETC, TCA cycle and β-oxidation were changed by the metformin treatment we assume that the stimulating effect of complex II or downstream should be ascribed to increased function, rather than expression, of one or more of these ETC complexes. This assumption is supported by persistence of the enhanced respiration through complex II or downstream in ETC, when mitochondrial respiration was expressed relative to mitochondrial content (CS activity).

The lack of a metformin-induced effect on respiration and mitochondrial enzymes in WT mice is in accordance with a recent study by Kane et al. [17] in rat muscle, although they measured on the combined effect of chronic (4 weeks) and acute (4 hours) effect of metformin treatment. In contrast, another study in rats by Suwa et al. [20] showed increased activities of both CS and HAD as well as increased expression of cytochrome c protein, after 2 weeks metformin treatment. The reason(s) for these discrepancies is not obvious, but could be related to the applied metformin dose. The study by Kane et al. used similar to the present study ∼300 mg kg^−1^ daily while the dosage was doubled in the Suwa et al. study to above 600 mg kg^−1^ daily. It is not possible to deduce whether the lack of an effect on these mitochondrial enzymes is due to the lower dose used. In the present study, metformin treatment is equivalent to most other *in vivo* rodent studies using 200–300 mg kg^−1^ daily [17], [46]–[49]. Although this dose is ∼7–10 times higher than recommended dose used in humans [50], [51] the literature reports comparable blood levels of metformin in humans and mice with the denoted treatment models [46], [50]. Therefore the dose used in the present and the study by Kane et al. probably elicits metformin levels comparable to a clinical situation. CS activity was measured in the predominantly glycolytic TA muscles in the present study whereas in the study by Kane et al. measurements were performed in the red oxidative part of gastrocnemious. Suwa et al. reported increased activity in both the red oxidative and white glycolytic part of gastrocnemious and also in soleus. These observations do not support a possible fibre type specific effect and a species specific difference is also doubtful since both Kane et al. and Suwa et al. studied rat muscles. Kane et al. used few animals (n = 4) compared to Suwa et al. (n = 8) and the present study (n = 12–17) which reduces the statistical power in the Kane study and thereby the risk of not detecting a potential difference. On the other hand, our study supporting the lack of an effect in the Kane et al. study is not biased by low statistical power. So the most obvious reason for the lack of a metformin effect is probably the lower metformin dose used by Kane et al. and us. This perspective is supported by newly published observations in muscles from patients with type 2 diabetes [7], [16]. Larsen et al. [16] found that metformin treatment of these patients did not affect complex I respiration and stated that clinical metformin treatment does not affect mitochondrial function in human skeletal muscle.

Unexpectedly we did see a stimulating effect of mitochondrial respiration in AMPK KD but not in WT muscles. In our view there is no obvious explanation for this finding, but we have some suggestions. If metformin, as shown in cell studies [11]–[14], [52], also inhibits complex I in these *in vivo* settings, there could be a potential redirection of substrate flux feeding into complex II and downstream in the ETC. In analogy to a physical exercise training regime this could induce an increased stress on mitochondrial ETC proteins downstream of complex I and a consequence could be adaptations to the stress leading to increased mitochondrial function which is also a common effect of exercise training [53], [54]. The reason why this only occurs in KD muscles could be a general reduction in mitochondrial protein expressions and activities. Meaning that a given stress stimuli on the muscle cell, e.g. metformin inhibition of complex I, is relatively higher the lower the initial mitochondrial content. On the other hand there are some observations in the study by Kane et al. [17] that could question these suggestions. First, in contrast to the *in vitro* cell studies, Kane et al. did not observe any inhibition of electron flux trough complex I four hours after *in vivo* metformin treatment with 320 mg kg^−1^. Consequently making it unlikely that we see an inhibition in the present study, where we divided the daily metformin dose into 2 feedings of 150 mg kg^−1^. On the other hand, it is possible that metformin-induced cellular stress in the more glycolytic TA muscle is amplified compared to the oxidative red gastrocnemious in the Kane study. Second, Kane et al. also missed a metformin effect in obese rats that like our KD mice had decreased mitochondrial capacity, based on CS activity. Accordingly, the argument of increased metformin-induced cell stress due to decreased mitochondrial capacity is not fully in line with the observations in the Kane et al. study, but again it is possible that susceptibility to mitochondrial reductions is different in glycolytic vs. oxidative muscles. An alternative hypothesis could be that AMPK, which as mentioned above function as a cellular energy gauge, somehow make the cell able to better cope with potential mitochondrial stress, induced by metformin. Thus, it is possible that the AMPK signaling pathway by an unknown mechanism counter regulates the metformin-induced inhibition of the oxidative metabolism, i.e. the ETC complex I, such that stress-induced adaptation of the ETC is absent. Observations of increased disturbances in muscle cell energy balance, indicative of greater cell stress, when genetic AMPK hampered mice are stressed by *in vivo* exercise support these thoughts i.e. greater metformin-induced cellular stress in AMPK KD muscles vs. WT muscles [55]–[58]. Additionally, such a connection was recently demonstrated in AMPK α1α2 double KO hepatocytes showing greater ATP decrease than wild type cells upon metformin incubation [52], [59].

Like in the AMPK defective mice, reduced mitochondrial content have been reported in skeletal muscles from patients with type 2 diabetes [3], [60]. Consequently, if diabetic muscles also have decreased AMPK expression and activity it could be suggested, based upon the speculations above, that metformin treatment of these patients enhances mitochondrial function. Although one study reports attenuated exercise induced AMPK activation in obese type 2 diabetic patients [61], and another study suggest that obesity has been shown to be inversely related to AMPK protein expressions in non diabetic humans [62], the majority of studies have shown no effect of type 2 diabetes on AMPK subunit protein expressions or basal and exercise induced activity [63]–[66]. Hence, it may be difficult to transfer the results from the present study of metformin-induced enhancement of mitochondrial function into clinical conditions, but further investigations on this topic in diabetic patients seem highly relevant.

Since a role for AMPK in mitochondrial function of skeletal muscle first time was elucidated for more than a decade ago by Winder et al. [30] numerous studies have expanded the knowledge in this field. Use of pharmacological AMPK activators [33], [40], [67]–[70], genetic models [32], [34], [55], [56], [58], [71]–[73] and combination of these [31], [33], [74] have substantiated AMPK as an important mitochondriogenic part. The kinase is involved both in maintenance of basal mitochondrial function and enhancement of mitochondrial enzymes and mitochondrial biogenesis upon increased AMPK activation. Hence, the decreased basal activities of CS and HAD, as key enzymes in the TCA cycle and β-oxidation, in the present study is a common observation reported in the AMPK KD strain [73], [75] and in comparable AMPK genetic model [31], [34], [55]. Also the decreased PDH protein expression, as a key regulator of carbohydrate metabolism, has been reported earlier in muscle of the AMPK whole body α2 knockout mice [55] and the muscle specific AMPK KD mice strain [75]. We recently reported decreased complex II and V protein expressions in the AMPK KD mice [75] but the present study is the first describing the expression profile of proteins representing all ETC complexes in the AMPK α subunit genetically manipulated model. Decreased expressions of these complexes have also been reported in AMPK β1β2 muscle knockout mice [34] and in LKB1 KO mice muscles, i.e. in muscles lacking the primary upstream AMPK kinase [56], [72]. Furthermore Lee et al. have looked at the individual activities of these complexes in muscles from a similar AMKP KD mouse strain as in the present study [57]. Their findings are partly in accordance with ours as they found decreased comlex I and V activities but no difference in the other complexes. The genotype difference on complex I and V persists when they are normalized to CS activity, in contrast to our finding with mitochondrial respiration through complex I and II. So Lee al. deduced that the genotype differences were not due to decreased mitochondrial content. This difference between the present study and Lee et al. could be due to methodological conditions and/or muscle type differences, since Lee et al. assayed the activities on homogenates of gastrocnemious while we measured respiration on whole muscle pieces from TA. Conversely, increased protein expression of complex I–IV has been shown in an alternative AMPK genetic model with an AMPK *activating* mutant of the regulatory γ3 subunit [32]. In that study mitochondrial respiration was also measured and in accordance with findings in the present study there were no genotype differences with electron flux through complex I and II when normalized to mitochondrial content. So, based on these studies it is a rather consistent finding that the AMKP system is important for regulation of oxidative phosphorylation via the ETC complexes. Although it is not fully resolved whether the genotype effect primarily is due to protein expression differences or functional conditions of the individual complex.

A possible limitation in our study is that not all endogenous AMPK activity is eliminated in the used AMPK transgenic model. Although we did not measure α1 or α2 associated activities directly in the present study based on the ACC Ser^221^ phosphorylation, an AMPK substrate, AMPK activity is reduced to ∼10% in AMPK KD muscles compared to WT muscle tissue. Our observations are in line with previous studies, using the AMPK KD mice, reporting α1 and α2 activities to be reduced with ∼90% [76]–[78] and ∼50% [76], [77], respectively, although α1 associated activity in one study was only moderately reduced (∼10%) [78]. Thus if AMPK is the only ACC Ser^221^ kinase then the ACC phosphorylation indicates that KD mice, in accordance with the previous studies, may have a small remaining AMPK activity. However, in our opinion the remaining AMPK activity in the AMPK KD mice does not affect our interpretation of the results and our suggestions.

We conclude that two weeks *in vivo* metformin treatment do not affect mitochondrial function or proteins in WT mice when applying a dose assumed to elicit plasma concentrations comparable to the clinical use of metformin. In contrast, in the mitochondrial deficient model of α2-AMPK KD mice, mitochondrial respiration is enhanced by metformin treatment. This metformin effect cannot be explained by regulation of mitochondrial key enzymes representing carbohydrate and fat metabolism or oxidative phosphorylation.
